# Specific and sensitive, ready-to-use universal fungi detection by visual color using ITS1 loop-mediated isothermal amplification combined hydroxynaphthol blue

**DOI:** 10.7717/peerj.11082

**Published:** 2021-03-18

**Authors:** Ilada Choopara, Yothin Teethaisong, Narong Arunrut, Sudaluck Thunyaharn, Wansika Kiatpathomchai, Naraporn Somboonna

**Affiliations:** 1 Program in Biotechnology, Faculty of Science, Chulalongkorn University, Bangkok, Thailand; 2 Department of Microbiology, Faculty of Science, Chulalongkorn University, Bangkok, Thailand; 3 Bioengineering and Sensing Technology Research Team, National Center for Genetic Engineering and Biotechnology, Pathum Thani, Thailand; 4 Faculty of Medical Technology, Nakhonratchasima College, Nakhon Ratchasima, Thailand; 5 Microbiome Research Unit for Probiotics in Food and Cosmetics, Chulalongkorn University, Bangkok, Thailand

**Keywords:** Loop-mediated isothermal amplification (LAMP), Hydroxynaphthol blue (HNB), Fungi, Diagnostic, Internal transcribed spacer (ITS), Ready-to-use

## Abstract

Being ubiquitous, fungi are common opportunistic pathogens to humans that can lead to invasive and life-threatening infections in immunocompromised individuals. Eukaryote-resembling cell membrane and filamentous branches make the fungal diagnosis difficult. This study therefore developed a ready-to-use ITS1 loop-mediated isothermal amplification combined with hydroxynaphthol blue (LAMP-HNB) for rapid, sensitive and specific colorimetric detection of universal fungi in all phyla. The ITS1 LAMP-HNB could identify every evolutionary phylum of fungi according to sequence analyses. We tested a total of 30 clinically relevant fungal isolates (representing three major human pathogenic phyla of fungi, namely Zygomycota, Ascomycota and Basidiomycota) and 21 non-fungal isolates, and the ITS1 LAMP-HNB properly identified all isolates, with a detection limit of as low as 4.6 ag (9.6 copies), which was identical to ITS1 and 18S rDNA PCR. The assays were also validated on the feasibility of point-of-care diagnostic with real food (dry peanuts, chili and garlics) and blood samples. Furthermore, the shelf life of our ready-to-use ITS1 LAMP activity (≥50%) was more than 40 days at 30 °C with 3–5% polyvinyl alcohol or glycerol additive. The results supported the ready-to-use ITS1 LAMP-HNB for simple detection of fungi contamination with high sensitivity in local and resource-constrained areas to prevent opportunistic fungal species infections.

## Introduction

Fungi are widespread and generally cause human, animal and plant diseases via opportunistic infections. Approximately 300 fungal species, mostly in phyla Zygomycota, Ascomycota and Basidiomycota, could be pathogenic to humans (Heitman, 2011; Gladieux et al., 2017; Janbon et al., 2019). For instances, Candida spp., Cryptococcus spp. and Aspergillus spp. that are found in environments represent common fungal pathogens to humans. Fungal diseases affected >1 billion humans and killed >1.5 million cases annually. For examples, over 10 million cases of fungal asthma (e.g. Aspergillus spp. and Cladosporium spp.), ∼1,000,000 cases fungal keratitis (Fusarium spp., Aspergillus spp. and Candida spp.), ∼3,000,000 cases chronic pulmonary aspergillosis (Aspergillus spp.), ∼700,000 cases invasive candidiasis (Candida spp.), and ∼223,100 cases cryptococcal meningitis complicating HIV/AIDS (human immunodeficiency virus and acquired immunodeficiency syndrome) (Cryptococcus spp.), were annually found (Hawksworth, 2001; Thomas & Kaliamurthy, 2013; Denning et al., 2014; Sloan & Parris, 2014; Denning et al., 2016; Bongomin et al., 2017; Pappas et al., 2018). The severity ranges from asymptomatic to mild, or life-threatening systemic infections (Hawksworth, 2001).

Contaminations of fungi and their toxins are of major concern, as they are ubiquitous and can survive extreme environments such as intense heat and dryness (Merino et al., 2019). Fungal eukaryote-like genomes and opportunistic pathogenesis from nonparasitic lifestyles with humans make the fungal diagnosis and treatment more complicated than bacteria (Casadevall, 2018). Hence, fungal diseases, although should be avoidable, have remained neglected from early accurate diagnosis and control. Designing universal fungi species diagnosis primers yet distinct from other eukaryote species require massive internal transcribed spacer 1, ITS1, sequences (>40,000 sequences) computer analyses and manual curation from scientists with experienced bioinformatics and LAMP backgrounds, and no scientist had ever developed a ready-to-use LAMP methods that covered all fungi species contamination. Subsequently, sensitive and specific detection of fungi, which themselves or their toxins cause food spoilage or could pose opportunistic infections and diseases to humans, using a ready-to-use assay at point-of-care and local settings for fungi sterility monitoring and control, for instances, in central surveillance laboratories, local clinics (i.e., sepsis), food markets (or at home) or manufacturers, is critical.

Traditional examination of fungi is by morphological characterization on selective culture media. Cultivation offers poor sensitivity and is time-consuming (extended time for fungal growth). Cultivation is also dangerous for pathogenic fungi, such as spores and toxins. These problems could be solved using nucleic acid detection by polymerase chain reaction (PCR) and loop-mediated isothermal amplification (LAMP) (Notomi et al., 2000). As PCR requires thermal cycler (3 h) and electrophoresis (0.5 h) instruments and times, LAMP utilizes a multi-strand displacement concept by the Bst DNA polymerase and 4-6 loop-intercalating primers to allow continuous amplification of target nucleotides at a single temperature in <1 h (no thermal cycler is required). Previous researches found the LAMP technique to be 10-100 folds more sensitive than PCR (Goto et al., 2007; Somboonna et al., 2018). In addition, the LAMP reagents can be combined with hydroxynaphthol blue (HNB), which represents a Mg^2+^ titrating dye in the LAMP reaction. During the nucleic acid amplification by LAMP, the concentration of Mg^2+^ is decreased, allowing visual color change of HNB as detected by naked eyes from violet (negative) to sky blue (positive). This replaces visualization of the LAMP product by agarose gel electrophoresis (GE), and has been used in several studies (Goto et al., 2009; Choopara et al., 2017). Nevertheless, currently available LAMP methods are specific to a single specie or genus, while fungal opportunistic pathogen species are diverse as afore-mentioned and mixed infection across species and genera in humans are documented, such as co-infection of Cryptococcus and Aspergillus in a healthy adult (Munaut, Van Hove & Moretti, 2011; Wang et al., 2018). Therefore, the present study firstly developed a ready-to-use, one-step ITS1 LAMP-HNB for simple and reliable (PCR-compatible) detection of any fungi contamination, identified assay sensitivity and specificity, tested in real dry food and clinical blood samples, and determined a shelf life of the ready-to-use, one-step reagents. We included pathogenic fungal species in specificity assay to confirm identification of human fungal pathogens, and our LAMP primers had been designed to flank a variable fungal species region where, in the future, scientists could design a species-specific nucleotide probe of specific species identification. Our LAMP primers located on the nuclear ribosomal internal transcribed spacers ITS1 (located between 18S and 5.8S rRNA genes) and ITS2 (5.8S and 28S rRNA genes) that have conserved sequences for merely fungi identification (Schoch et al., 2012), not on a 18S rRNA gene that is common to all eukaryotes.

## Materials and Methods

### Microbial strains, culture and crude DNA extraction

Microorganisms used in this study included 30 fungal isolates (22 species in phyla Zygomycota, Ascomycota and Basidiomycota) and 17 bacterial species (in classes Bacilli, Antinobacteria, Chlamydiae, Gammaproteobateria and Betaproteobacteria) (Table S1). All fungi were cultured in potato dextrose agar (20 g dextrose, 200 g potatoes infusion form, and 15 g agar, per 1 L water). Bacteria were cultured in blood agar (trypticase soy agar enriched with 5% human blood). Crude DNA extraction was performed by resuspending isolate colonies of fungi (or bacteria) in 20 μL of Tris-EDTA buffer (10 mM Tris Base and 1 mM EDTA, in pH 8.0), boiling at 95 ^∘^C for 15 min (fungi) or 10 min (bacteria), and placed on ice prior to centrifugation at 11,772 × g for 10 min. The supernatant was used as DNA template. Quality and concentration of crude DNA extracts were determined by A260/A280 and A260 nanodrop spectrophotometer, respectively.

### LAMP primer design for universal fungi detection

Nucleotide sequences of ITS1 gene from >40,000 fungal species covering all four phyla of kingdom fungi were downloaded from GenBank (http://www.ncbi.nlm.nih.gov/genbank/), and multiple sequence alignments were performed using ClustalW (http://www.megasoftware.net/). Six degenerate primers were manually designed to locate on eight conserved regions on ITS1 and flank variable region among fungal species (https://primerexplorer.jp/e/) (Table 1). The specificity and coverage of each primer was checked using BLASTN (Camacho et al., 2009).

**Table 1:** Primer sequences for ITS1 LAMP-HNB and 18S rDNA PCR.

**Method**	**Primer names**	**Sequences (5′→3′)**
LAMP	ITS1_FIP (F1c/F2)	TCCGCAGGTTCACCTAC-TTT-YGARAAGYTCGTCAAAC
	ITS1_BIP (B1c/B2)	CTTTCAACAAYGGATCTCTTG-TTT-ACATTACTTATCGCATTTCG
	ITS1_F3	GATTGAATGGCTYRGTGA
	ITS1_B3	AAKRTGCGTTCAAAGATT
	ITS1_LF	ACGGARACCTTGTTACG
	ITS1_LB	CTSGCATCGATGAAGAA
PCR	Euk1A	CTGGTTGATCCTGCCAGT
	Euk516A	ACCAGACTTGCCCTCC

**Notes.** *Y* = *C*∕*T*; *R* = *A*∕*G*; *K* = *G*∕*T*; *S* = *C*∕*G*

### ITS1 LAMP-HNB

LAMP-HNB reaction (15 μ L) comprised 0.2 μM each of primers F3 and B3, 1.6 μ M each of FIP (F1c/F2) and BIP (B1c/B2), 1.4 μ M each of LF and LB, 1.4 mM dNTP (SibEnzyme Ltd., Novosibirsk, Russia), 1 M betaine (Sigma–Aldrich, St. Louis, MO, USA), 6 mM MgSO4, 8 U Bst DNA polymerase (large fragment) (New England Biolabs Inc., Beverly, MA, USA), 1 × ThermoPol^TM^ Reaction Buffer (New England Biolabs Inc.), 133μ M HNB (Sigma-Aldrich, St. Louis, MO, USA), and 100 ng DNA (unless specified). The reaction conditions (shortest incubation time with highest sensitivity and correct specificity) were optimized using temperatures of 55, 58, 60, and 63 ^o^C, and incubation periods of 20, 25, 30, 40 and 45 min. *A. flavus* MSCU0850 was used as template for finding optimized isothermal condition and a limit of detection of ITS1 LAMP-HNB. Result was analyzed by naked eyes from violet (negative, ∼550–575 nm) to sky blue (positive, ∼650 nm). On 1.75% agarose gel electrophoresis at 100V (GE), the LAMP products appeared as intercalating bands of various base pair (bp) sizes.

### ITS1 and 18S rDNA PCR-GE

PCR reaction (25 μL) comprised 0.3 μM each of forward and reverse primers (Table 1: ITS1_F3 and ITS1_B3 for ITS1, and Euk1A and Euk516A for 18S rRNA gene) (Wang et al., 2014), 12.5 μL EmeraldAmp^®^ GT PCR Master Mix (TakaRa Bio Inc., Shiga, Japan), and 100 ng DNA (unless specified). The thermal cycling condition was 94 ^∘^C 4 min followed by 35 cycles of 94 ^∘^C 45 s, 50 ^∘^C 1 min and 72 ^∘^C 1.5 min, and final extension at 72 ^∘^C 10 min. The PCR product was analyzed by GE.

### Comparison of specificity and sensitivity (detection limit) among ITS1 LAMP-HNB, ITS1 PCR-GE and 18S rDNA PCR-GE

The specificities of the assays were determined with representative fungi (positive) and bacteria (negative) isolates (listed in Table S1). Additional negative controls included crude extracted DNA from human (Homo sapiens) (Somboonna et al., 2018; study was approved by the Institutional Review Board of Buddhachinaraj Phitsanulok Hospital (101/54) and the Ethics Committee for Research in Human Subjects of the Department of Disease Control, Bangkok (FWA00013622)), pork (*Sus scrofa*), chicken (*Gallus gallus*) and fish (*Pangasius hypophthalmus*) meats from local supermarket in Bangkok, Thailand. Crude DNA extraction from additional negative controls was performed by resuspending a sample in 20 μL of Tris-EDTA buffer, boiling at 95 ^∘^C for 15 min, and placed on ice prior to centrifugation at 11,772 × g for 10 min. The supernatant was used as DNA template. The detection limits were carried out using a series of 10-fold dilutions of ITS1 and 18S rDNA of *A. flavus* MSCU0850 in the range of 9.6 ×10 ^6^ to 0.96 (equivalent to 4.6 pg to 0.46 ag) and 9.71 ×10 ^6^ to 0.97 copy numbers (equivalent to 5.9 pg to 0.59 ag), respectively. The results of LAMP-HNB were monitored via naked eyes and confirmed by UV-vis spectra measurement and GE. The performance of ITS1 LAMP-HNB was compared in parallel with ITS1 PCR-GE (using primers corresponding to the same nucleotide region, which were ITS1_F3 and ITS1_B3 in Table 1) and 18S rDNA PCR-GE. Noted that because LAMP for universal fungi had none reported, this assay compared with 18S rDNA PCR-GE from established protocols (Wang et al., 2014). Further, the assay performance of the ITS1 LAMP-HNB was validated using real dry food samples (five samples each of peanuts, chili and garlics from local markets) with and without artificial microbial contamination, and a healthy human blood sample in EDTA buffer (Ethical Committee of the Faculty of Medicine, Chulalongkorn University, E.C.NO.654/60) with artificial microbial contamination, and was compared with PCR-GE. Each microbial isolate was grown in pure culture, then centrifuged and 1 pg microbial isolate pellet was used for artificial microbial contamination. For crude DNA extraction, 0.3 g food sample (or 0.1–0.3 mL blood sample) was mashed and transferred to an empty one mL eppendorf tube, and the sample was irradiated in a microwave oven at ≥ 800 W 5 min, along a presence of water-containing beaker during the irradiation to avoid the microwave damage. Next, 300 mL TE (10 mM Tris-HCl pH 7.5, 1 mM EDTA) was added to resuspend, vortexed, centrifuged at 11772 × g 1 min, and the supernatant was used as template for LAMP-HNB and PCR-GE (Ferreira, Silva & Panek, 1996).

### Effect of preserving additive on shelf-life of ready-to-use ITS1 LAMP-HNB reagents

Choices of preserving additives and concentrations (3% and 5% glycerol, and 3% and 5% polyvinyl alcohol (PVA)) were added to the whole ITS1 LAMP reagents including Bst DNA polymerase (representing “ready-to-use LAMP reagents” owning to their containment of every ingredient except HNB and DNA template of interest), and the ready-to-use reagents were stored at 30, 50 and 80 ^∘^C for a period of 2-30 days. In brief, the methods of identifying shelf-life of the ready-to-use LAMP reagents with each preserving additive were followed established protocols (Labuza, 1984; Khalid et al., 2011). A fluorescent DNA dye, Novel Juice (BIO-HELIX, Keelung, Taiwan), was used to track the level of LAMP reaction activity (the amplified product) at each time series of storage. The florescent intensity was analyzed by ImageJ software (https://imagej.nih.gov/ij/download.html), and was proportional to the amount of the LAMP products. Subsequently, the reduction in the fluorescent intensity could define a percentage of LAMP activity remaining. Three independent replicates were performed and measured the percent remaining activity per time point to obtain the average and standard deviations. The remaining percentages of the LAMP activity against storage times (days) for each choice of preserving additives and concentrations were plotted, and derived a regressive equation with storage days and remaining % LAMP activities as X and Y variables, respectively, along the goodness-of-fit index (*R*^2^).

## Results

### Primer design and optimal isothermal condition for ITS1

**LAMP-HNB**

Regions for 6 LAMP primer sequences were designed based on conserved regions among fungal species following ITS1 sequence alignment analysis. Figure S1 displayed minor nucleotide variation among these conserved primer regions, and degenerate primers (Table 1) were thereby designed to cover all possible sequences of fungal species (Iserte et al., 2013). The ITS1 region between 18S and 5.8S rRNA genes is a specific target for universal fungi, and the variable region in ITS1 between our designed primers ITS1_F1 and ITS1_BIP is rather specific to allow identification of fungi at a species level (Iwen, Hinrichs & Rupp, 2002; Blaalid et al., 2013; Yang et al., 2018).

Following a range of reaction temperatures and time durations, the optimal isothermal condition for our LAMP-HNB reaction (shortest incubation time with highest sensitivity and correct specificity, Fig. S2) was found at 58 ^∘^C and 40 min, using crude fungal DNA lysate from simple boiling at 95 ^∘^C for 15 min (details in Materials and Methods) as a template.

### Specificity and detection limit of ITS1 LAMP-HNB

**Figure 1:**
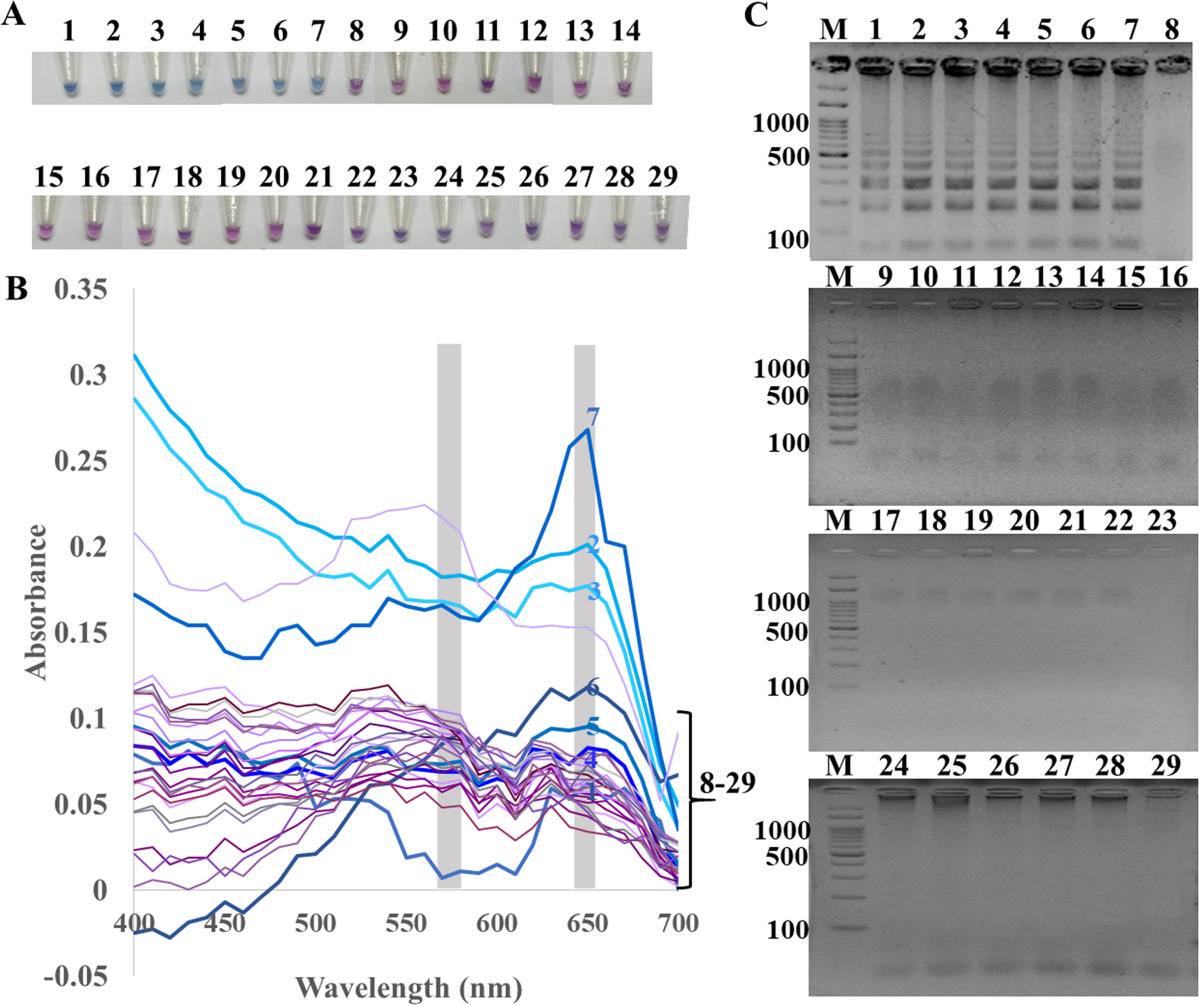
Specificity of ITS1 LAMP analyzed by (A) visual color readout using HNB, whereby a color change from violet to blue indicates a positive reaction, (B) UV-vis spectra, and (C) GE. Positives (numbers 1–7) included crude extracted DNA from *Candida albicans*, *Aspergillus carbonarius*, *Aspergillus flavus*, *Curvularia lunata*, *Rhodotorula mucilaginosa*, *Rhizopus oligosporus*, and* Ustilago esculenta*. Negatives (numbers 8–29) included crude extracted DNA from human (*Homo sapiens*), swine (*Sus scrofa*), chicken (*Gallus gallu* s), fish (*Pangasius hypophthalmus*), and bacteria *Streptococcus pneumonia*, *Proteus mirabilis*, *Staphylococcus aureus*, *Shigella* sp., *Salmonella* sp., *Corynebacterium* sp., *Burkholderia cepacia*, *Acinetobacter baumannii*, *Klebsiella pneumoniae*, *Pseudomonas aeruginosa*, *Enterococcus* sp., *Staphylococcus epidermidis*, *Staphylococcus saprophyticus*, *Neisseria gonorrhoeae*, *Chlamydia trachomatis*, *Escherichia coli*, *Salmonella typhimurium*, and sterile water. In (C), M represents GeneRuler™ 100 bp plus DNA ladder (Invitrogen).

**Figure 2:**
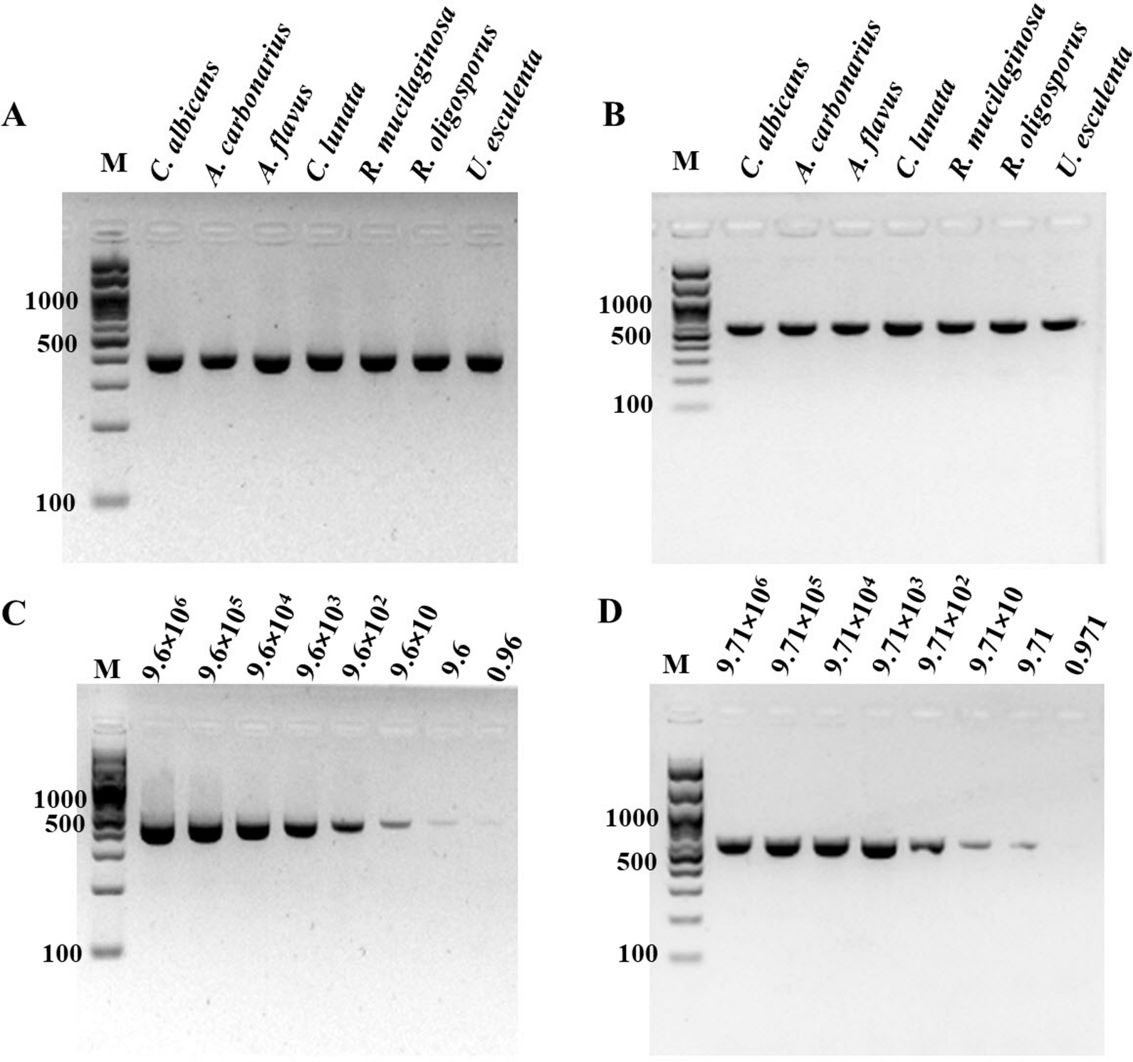
Specificity and sensitivity (limit of detection) of (A and C) ITS1 and (B and D) 18S rDNA PCR-GE. For (C) and (D), *A. flavus* MSCU085 was used as template. M represents GeneRuler™100 bp plus DNA ladder (Invitrogen).

The specificity of ITS1 LAMP-HNB was validated using 7 representative fungal species and 21 non-fungi species (Fig. 1A). Additional specificity assay on 23 fungal isolates with clinically relevance in human or foodborne diseases were also demonstrated (Fig. S3, e.g., Epidermophyton floccosum, Trichophyton rubrum, Cryptococcus neoformans, Microsporum gypseum, Flusarium sp., Phialophora verrucosa, Penicillium marneffei (also known as Talaromyces marneffei) and Candida albicans). Together, all 30 clinically relevant fungal isolates (accounting for 22 fungal species) were properly tested positive using ITS1 LAMP-HNB. The proper color and amplicon results were confirmed by UV-vis spectra (Fig. 1B) and GE (Fig. 1C), respectively. It is important to note that the positive color all had a ratio of A650/A580 >1.00, while the ratio <1.00 corresponded to negative results (Goto et al., 2009; Choopara et al., 2017). The PCR targeting ITS1 and 18S rRNA gene showed identical results (Figs. 2A and 2B), heightening the reliability of our ITS1 LAMP-HNB reaction protocols (compatible to PCR assays) and primers (compatible to other universal eukaryotic primers Euk1A and Euk516A for 18S rDNA) (Wang et al., 2014).

**Figure 3:**
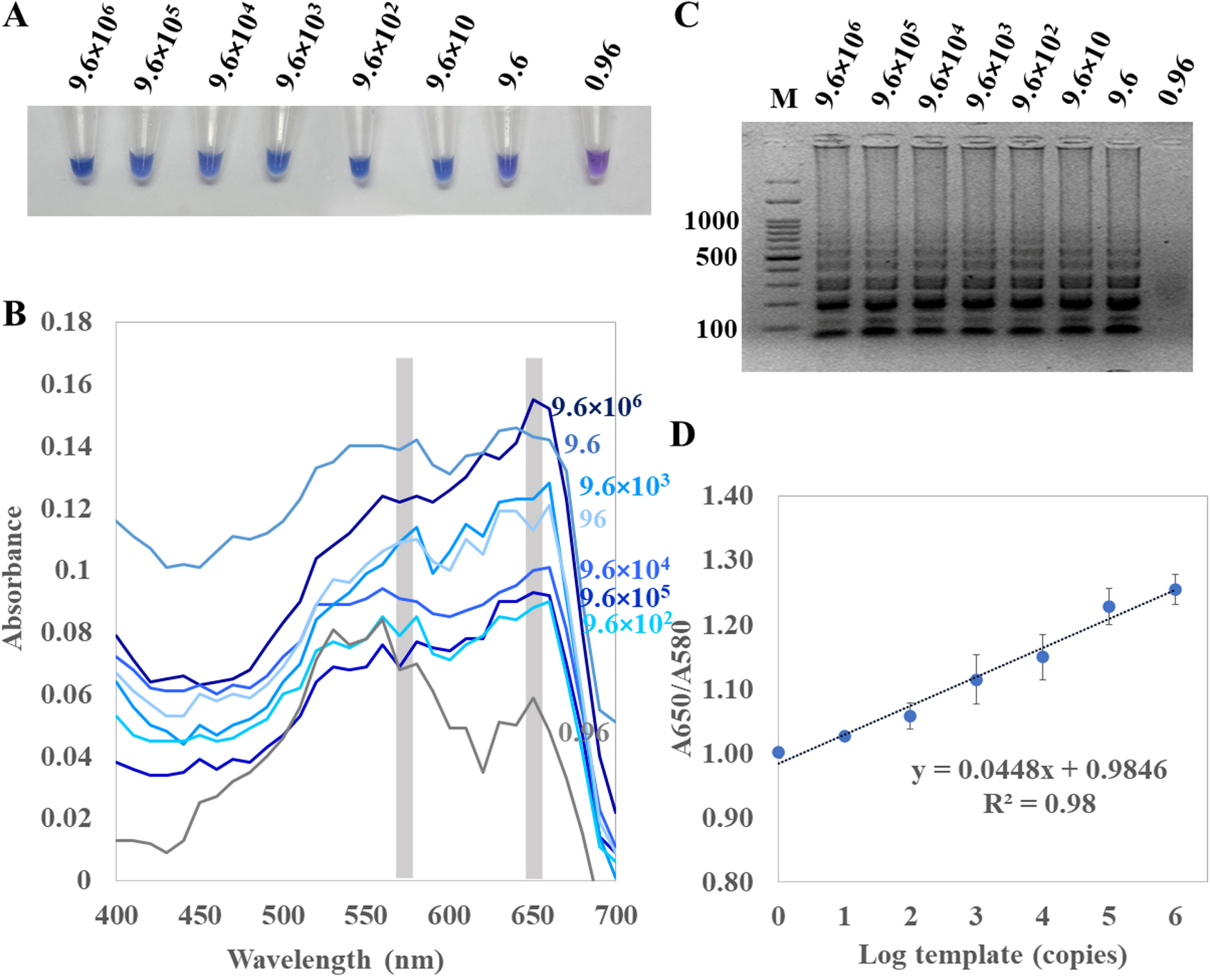
Limit of detection of ITS1 LAMP analyzed by (A) visual color readout, (B) UV-vis spectra and (C) GE; and (D), a regressive equation for quantitation of template (copies, X variable) that was generated from a ratio of A650/A580 of the UV-vis spectra. (*Y*-axis) against log_10_ concentration of template (*X*-axis), resulted in the quantitation of template (copies) with reliable *R*^2^ of 0.98. In (A) to (C), the limit of detection was found 9.6 copies of ITS1 (equivalent to 4.6 ag). *A. flavus* MSCU085 was used as template, and in (C), M represents GeneRuler™  100 bp plus DNA ladder (Invitrogen).

For determination of the detection limit of ITS1 LAMP-HNB, the minimum concentration that could be detected by naked eyes (Fig. 3A) consistent with UV-vis spectra (Fig. 3B) and GE (Fig. 3C), was found to be as low as 9.6 copies (equivalent to 4.6 ag) of ITS1. This limit of detection was compatible with ITS1 and 18S rDNA PCR-GE (Figs. 2C and 2D). The results of our ITS1 LAMP-HNBs were consistent, and thus the data from independent triplicates were able to generate the confident linear regression (goodness-of-fit measurement, *R*^2^ = 0.98) for quantitative detection of a template with an unknown concentration from the ratio of A650/A580 by UV-vis spectra (Fig. 3D: *Y* = 0.448*X* + 0.9846, where *Y* is A650/A580 and *X* is log_10_ copies).

### Comparative performances between ITS1 LAMP-HNB and PCR-GE assays on dry food and clinical blood samples detection

**Figure 4:**
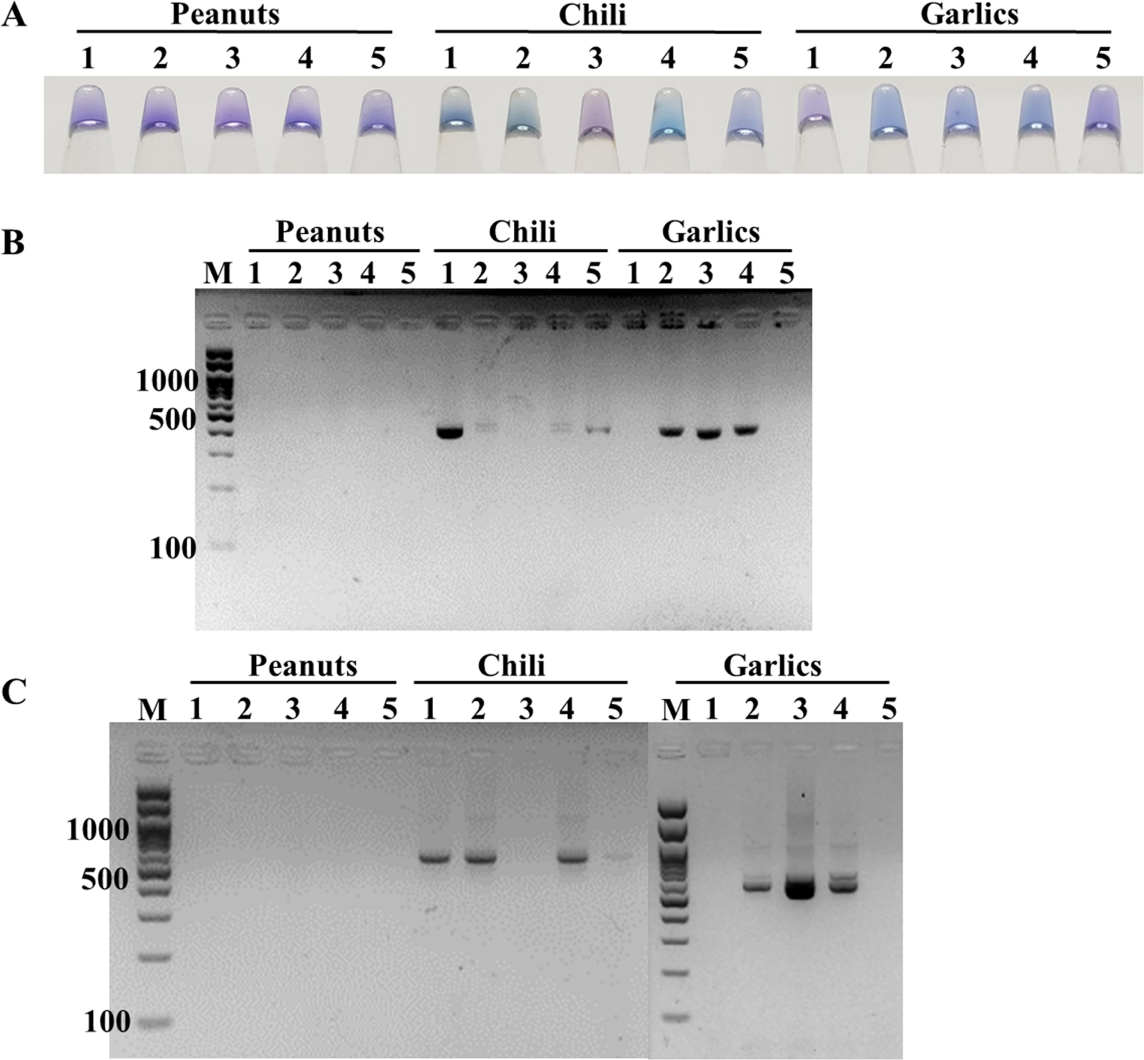
Comparative performance of (A) ITS1 LAMP-HNB, (B) ITS1 PCR-GE and (C) 18S rDNA PCR-GE, for detecting real dry food samples (peanuts, chili and garlics).

The feasibility of the point-of-care ITS1 LAMP-HNB assay was evaluated, starting from 6-min crude DNA lysis to LAMP-HNB and visual color readout, in comparison to ITS1 and 18S rDNA PCR-GE assays. The reasons we compared with PCR-GE were that we could use our ITS1 LAMP primers (Table 1, ITS1_F3 and ITS1_B3) for ITS1 PCR, allowing parallel comparison with no bias on primer region selection, and that the standard methods for nucleotide amplification and amplicon visualization are PCR followed by GE. A total of 15 real dry food samples that might contain fungi contamination (peanuts, chili and garlics) were tested. Three independent experiments were performed. Based on the ITS1 PCR-GE as the standard assay (also the 18S rDNA PCR-GE), our ITS1 LAMP-HNB assay showed identical findings (Fig. 4). Hence, triplicate analyses of the 15 dry food samples by the ITS1 LAMP-HNB assay showed 100% sensitivity (7/7 positives) and specificity (8/8 negatives). Moreover, artificially contaminated dry food samples were performed to confirm the reliability of our ITS1 LAMP-HNB assay by adding *A. flavus* MSCU0850 culture to autoclaved (sterile) peanut, chili and garlic; meanwhile adding sterile water in replace of *A. flavus* MSCU0850 to autoclaved dry food sample (negative contamination) showed negative result (Figs. S4A and S4B). Our ITS1 LAMP-HNB was also evaluated its testing reliability on a clinical blood sample from a healthy (no infection) subject, without (as negative control) and with artificially microbial contamination by *A. flavus* MSCU0850 and *A. flavus* MSCU0850 mixed *E. coli* (positive tests) and *E. coli* (negative test) (Figs. S4C and S4D). Noted the visual color change of HNB may sometimes be unclear because of low presence of fungi or a color bias from an original sample, e.g., in Fig. 4A and Fig. S4, the crude extracted DNA from food samples had creamy white or reddish color.

### Preserving additive to extend shelf life of ready-to-use ITS1 LAMP-HNB

**Figure 5:**
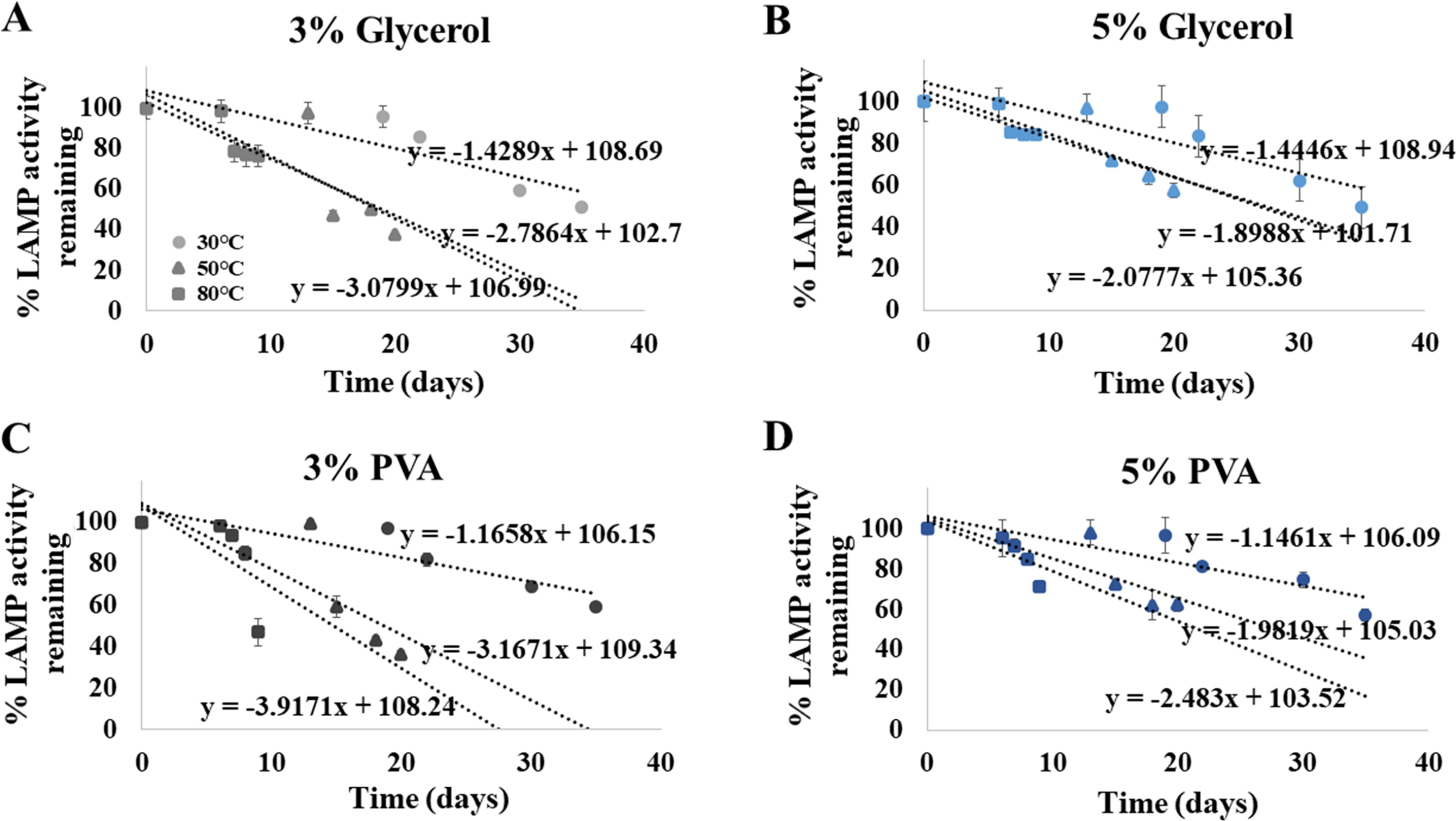
Efficiency of the ITS1 LAMP activity (*Y*-axis), as reported by fluorescent DNA-dye intensities, along different storage days (*X*-axis) and temperatures (30, 50 and 80°), using (A) 3% glycerol, (B) 5% glycerol, (C) 3% PVA, and (D) 5% PVA as preserving additive into the ready-to-use ITS1 LAMP reagents. The data in (A) to (D) were from independently triplicate experiments.

Shelf life extension of our ready-to-use ITS1 LAMP-HNB reagents was attempted by adding 3% or 5%, of glycerol or PVA. After an addition of each preserving additive, the same detection limit (4.6 ag) was validated. Figures 5A-5D demonstrated that the higher concentration (5%) and the type of the preserving additive (PVA, instead of glycerol) both affected the shelf life of the ready-to-use ITS1 LAMP reagents. The shelf life at 90% remaining LAMP activity under 30 ^∘^C storage using 3% glycerol, 5% glycerol, 3% PVA or 5% PVA as a preserving additive, was found to be 13.08, 13.11, 13.853 and 14.038 days, respectively. The ready-to-use ITS1 LAMP reagents with 3–5% glycerol and 3–5% PVA showed ≥ 50% remaining activity until 40.80–41.07 and 48.16–48.94 days, respectively (Table S2). The derived equations all had >96% goodness-of-fit, which meant that smaller than 5% variation to the estimate was only possible.

## Discussion

Fungi are ubiquitous and represent one common cause of food spoilage and opportunistic human pathogens. Approximately 300 fungal species have been reported, hence to be able to detect all opportunistic fungal pathogens via portably rapid DNA assay as effective as standard molecular technique like PCR is none available owning to difficulty in massive (>40,000) sequences analyses for 6 LAMP primers design and modern technique of LAMP. Our ready-to-use ITS1 LAMP-HNB assay, including steps from crude DNA extraction of original food and clinical samples until color result readout, is the first that could specifically detect all fungal species at high sensitivity (limit of detection) compatible to PCR. Our ready-to-use ITS1 LAMP-HNB assay is simple without special instrument (i.e., thermal cycler and electrophoresis), and takes only 6 min for crude DNA extraction and 40 min for reaction time (<1 h total). The crude DNA extraction method replaced a more time- and cost-consuming commercial DNA extraction and purification kit, and in part supported a ready-to-use assay (Zhang et al., 2010). The advantage of ITS1 over 18S rDNA nucleotide regions for fungi detection was that the 18S rDNA sequence is not sufficiently variable enough to identify fungi to genus and species levels, while the ITS1 sequence is more variable and could completely classify fungi to genus and species levels (Köhsler et al., 2006; Liu et al., 2015). Thus, using the ITS1 LAMP primers allows later nucleotide sequence probe detections specific for any fungal genera (or species). A specific nucleotide sequence probe, such as in Somboonna et al. (2018), may be designed as a second step to selectively identify severely virulent species, for instance. Further, the ability to directly quantitate the color readout into copies of fungi via just a spectrophotometry reading minimizes extra cost and time, and supports local detection where laboratory equipments are limited.

Overall results from 51 positive and negative references as well as real food and clinical samples evaluation demonstrated that our ready-to-use, 1-step ITS1 LAMP-HNB offered high specificity and sensitivity for fungi detection, and the assay was rapid (<1 h) and simple requiring only a water bath (or a heat pad) of 58 ^∘^C (Notomi et al., 2000; Somboonna et al., 2018). In addition, this assay gave negative results to common (uncontaminated) food, clinical and environmental (e.g., air, drinking water; Fig. S5) samples. For quantitation, a spectrophotometer for A650 and A580 absorbance readings was required. However, some obscured color results, i.e., a color bias from original food sample, might be solved by a selection of a different dye color, such as a pH-sensitive neutral red or phenol red dye, that the release of hydrogen ions in amplifying LAMP products result in a final acidic pH (Tanner, Zhang & Evans, 2015; Poole et al., 2017), or a spectrophotometry reading. Yet, using neutral red or phenol red dye will have a color bias problem from chili and blood samples, for examples. In replace of HNB and a visual color bias problem from original samples, the ITS1 LAMP product may be GE confirmed (requires agarose gel electrophoresis apparatus), or stained with a fluorescent nucleotide dye, such as SYBR Green or styryl, but this dye requires a UV lamp for color visualization and an additional step of fluorescent dye staining after LAMP reaction (Srimongkol et al., 2020).

Furthermore, our study included method for extending shelf life of the ready-to-use ITS1 LAMP-HNB reagents. Choices of concentrations and preserving additives (3% or 5%, of glycerol or PVA) had been selected based on established reports (Basiak, Lenart & Debeaufort, 2018; Syed, Garg & Sarkar, 2018), and 5% PVA showed the relatively longest shelf life. More than 5% PVA or glycerol were not tested because too high concentration of these preserving additives could deteriorate the LAMP efficiency (Tzeling & Yean, 2016; Seok et al., 2017). Moreover, HNB was reported ineffective beyond >7 days of storage (Brittain, 1978; Goto et al., 2009). Thus, when the reagents will be stored for more than 7 days, HNB was added in the ready-to-use LAMP reagents later at the time of diagnosis.

## Conclusions

Our ready-to-use ITS1 LAMP-HNB assay (Thailand petty patent no. 1803002277, 2 October 2018) is specific to universal fungi species and sensitive (9.6 copies, or 4.6 ag) as high as PCR-GE. The assay time is rapid (<1 h), and the method is simple without special instrument (i.e., thermal cycler and electrophoresis) for end-point users. Hence, this assay represents a prototype detection for any presence of fungi in food, blood (e.g., fungal sepsis or drug-resistant fungal sepsis), or in any samples at point-of-care or local settings. However, for food sample that fungi may present as starter culture, this assay may be omitted, or else used as the monitoring assay to screen food processing and food product steps to satisfy a general food quality control that does not allow fungal presence. The color readout of our ITS1 LAMP-HNB is clear, and with A650/A580 spectra reading, the presence of fungi can be quantitated. Further, the studied preserving additive allowed storage of these ready-to-use ITS1 LAMP reagents up to 14 and 49 days at 30 ^∘^C for 90% and 50% remaining LAMP activity, respectively.

##  Supplemental Information

10.7717/peerj.11082/supp-1 Supplemental Information 1 Relative abundances of each nucleotide (A, T, C and G) along sequences of LAMP primers ITS1_FIP (F1c/F2 regions), ITS1_F3, ITS1_BIP (B1c/B2), ITS1_B3, ITS1_LF and ITS1_LB *Y*-axis represents position along each primer sequence, and *X*-axis represents a possibility of A, T, C and G.

10.7717/peerj.11082/supp-2 Supplemental Information 2 Incubation periods (20, 25, 30 and 40 min) and temperatures (55, 58, 60, and 63° C) of ITS1 LAMP-GE, using *A. flavus* MSCU085 DNA as template

10.7717/peerj.11082/supp-3 Supplemental Information 3 Additional specificity assay of ITS1 LAMP analyzed by (A) visual color readout using HNB, whereby a color change from violet to blue indicates a positive reaction, and (B) GE Templates are crude extracted DNA from *E. floccosum, T. rubrum, C. neoformans, M. gypseum, Flusarium* sp.*, P. verrucosa, P. marneffei*, sterile water (negative), *C. krusei* (isolate 1), *C. albicans* (isolate 1)*, C. glabrata* (isolate 1), *Histoplasma* sp., *Curvularia* sp., *C. krusei* (isolate 2)*, C. glabrata* (isolate 2)*, C. parapsilosis, C. dubliniensis, C. guilliermondii, C. albicans* (isolate 2), *C. albicans* (isolate 3), *C. albicans* (isolate 4), *C. tropicalis* (isolate 1), *C. tropicalis* (isolate 2) and *C. tropicalis* (isolate 3), respectively.

10.7717/peerj.11082/supp-4 Supplemental Information 4 LAMP-HNB and LAMP-GE testings of artificially contaminated (A and B) dry food and (C and D) blood samples (A and B) artificially contaminated dry food samples that were tested negative in Fig. 4 by adding 1 pg of *A. flavus* MSCU0850 culture. (C and D) blood from a healthy volunteer by adding 1 pg each of *A. flavus* MSCU0850, *E. coli*, or *A. flavus* and *E. coli* cultures, and sterile water (as negative), respectively.

10.7717/peerj.11082/supp-5 Supplemental Information 5 LAMP-HNB (left image) and LAMP-GE (right image) testings of three air and three drinking water samples For air sampling, cotton swab was hold in air in laboratory for 1 min before used in crude DNA extraction.

10.7717/peerj.11082/supp-6 Supplemental Information 6 Raw data

10.7717/peerj.11082/supp-7 Supplemental Information 7 List of microorganisms used in this study

10.7717/peerj.11082/supp-8 Supplemental Information 8 Resolved Fig. 5 equations for days at 50, 75 and 80% ITS1 LAMP activity at 30° C storage, using different preserving additives

## Additional Information and Declarations

### Data Availability

The following information was supplied regarding data availability: Raw data, including agarose gel electrophoretic images and color change records by photographs and spectrophotometry scan, are available in the Supplementary Files and Figs. 1– 5.
