# Supplementary figures and images for: Specific and sensitive, ready-to-use universal fungi detection by visual color using ITS1 loop-mediated isothermal amplification combined hydroxynaphthol blue
Source: PeerJ. 2021 Mar 18;9:e11082. doi: 10.7717/peerj.11082 (PMC7982077; doi:10.7717/peerj.11082)

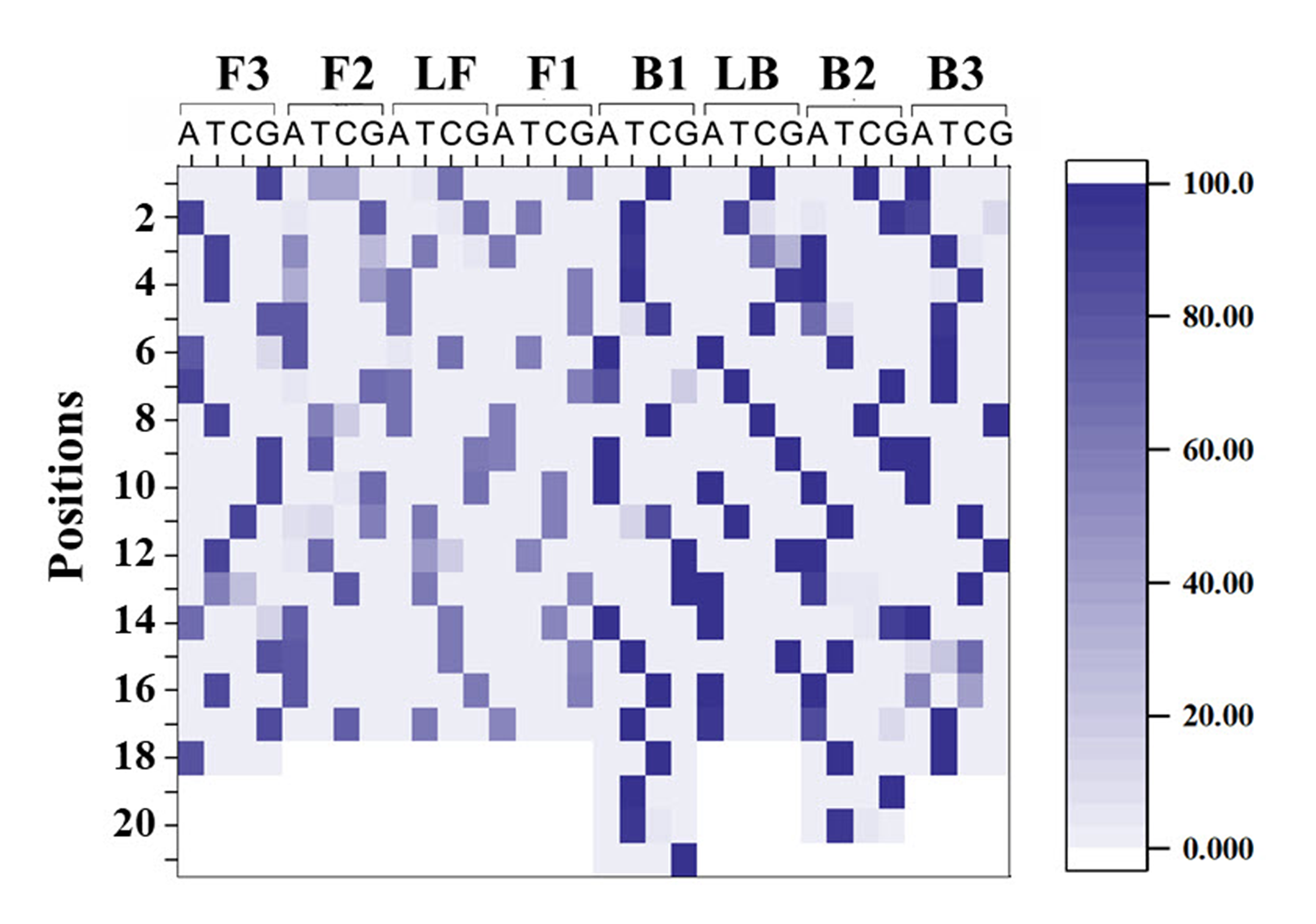

Supplement: Supplemental Information 1 — Y-axis represents position along each primer sequence, and X-axis represents a possibility of A, T, C and G. [file peerj-09-11082-s001.png]

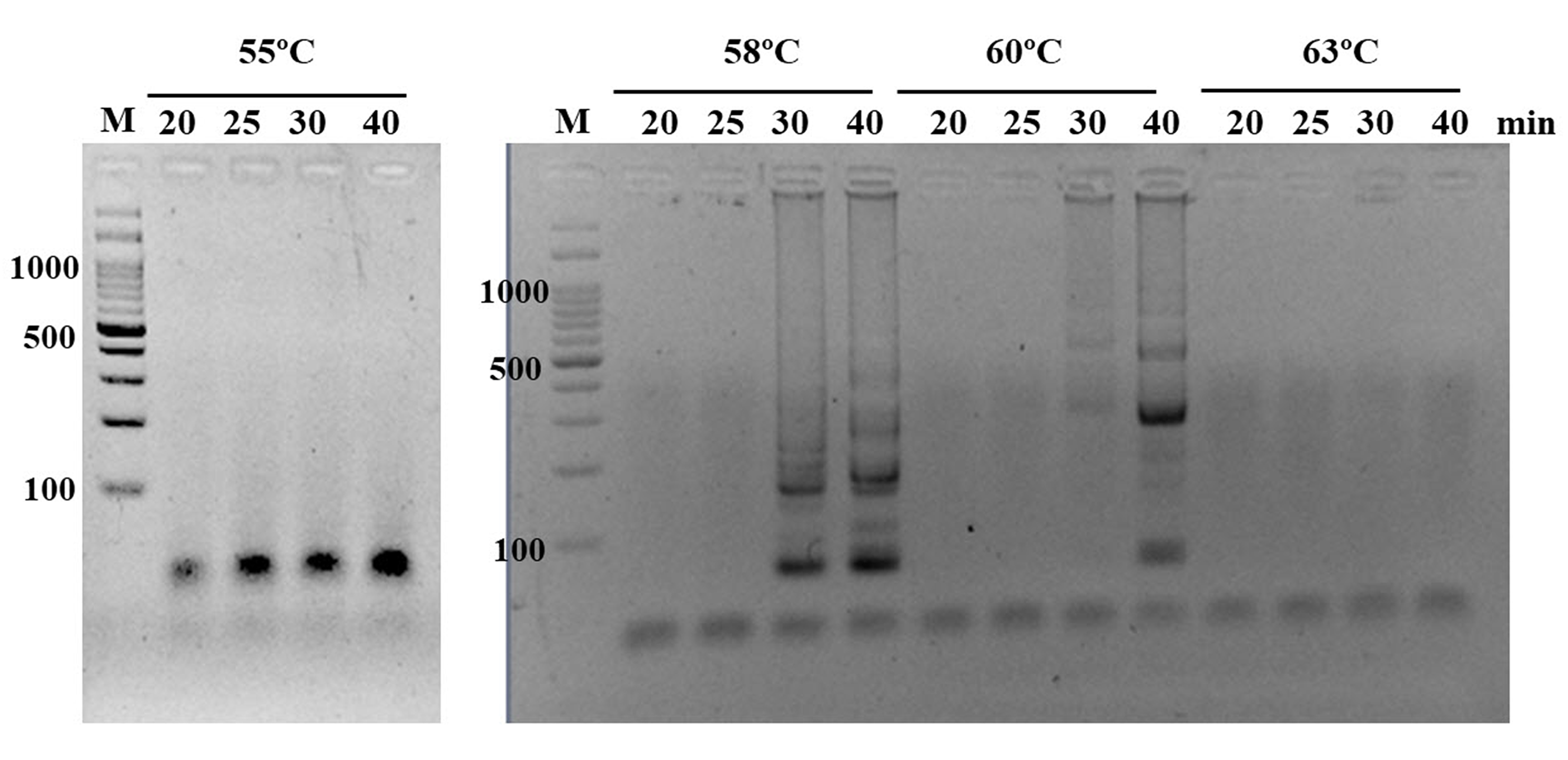

Supplement: Supplemental Information 2 [file peerj-09-11082-s002.png]

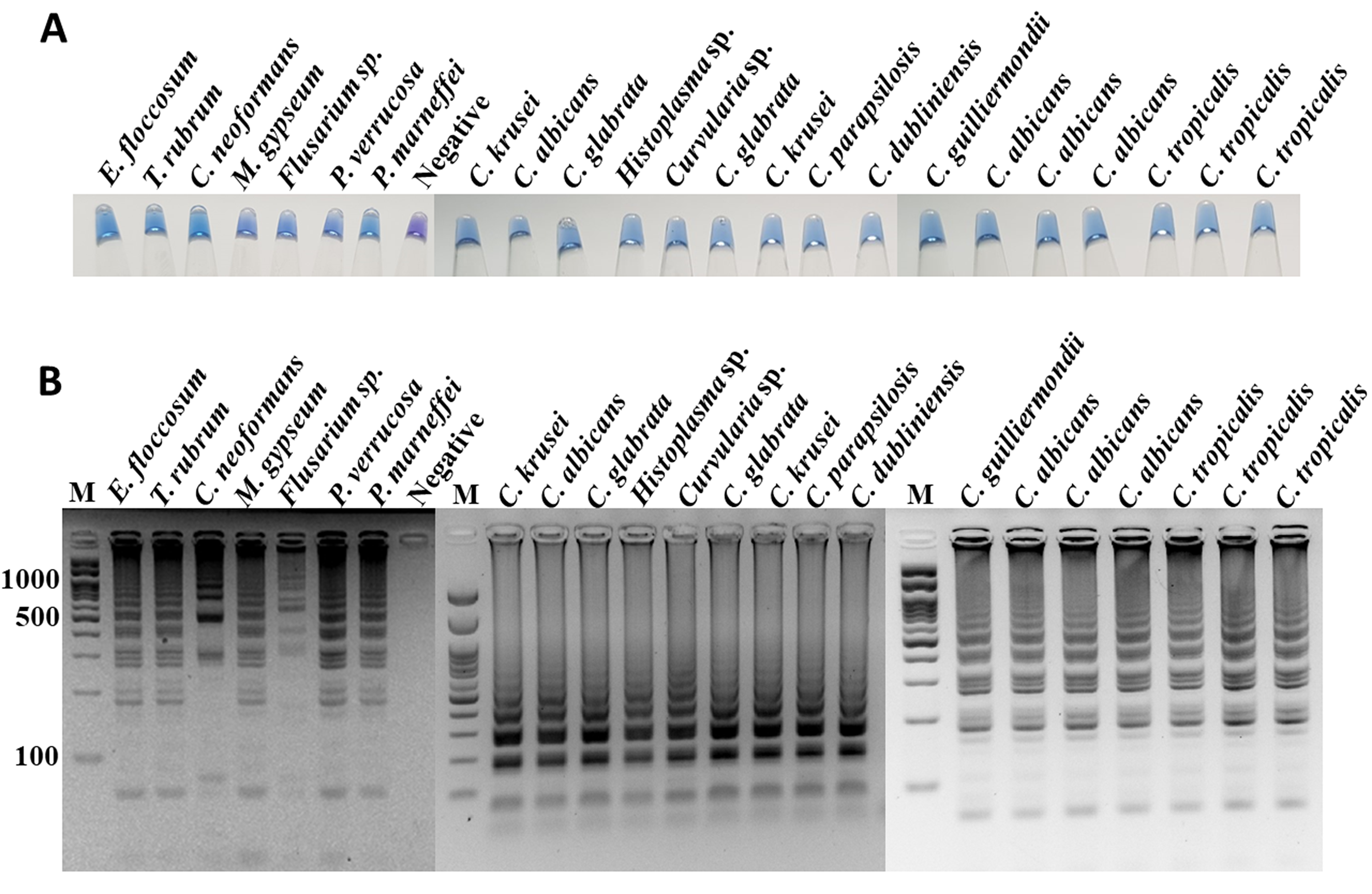

Supplement: Supplemental Information 3 — Templates are crude extracted DNA from E. floccosum, T. rubrum, C. neoformans, M. gypseum, Flusarium sp., P. verrucosa, P. marneffei, sterile water (negative), C. krusei (isolate 1), C. albicans (isolate 1), C. glabrata (isolate 1), Histoplasma sp., Curvularia sp., C. krusei (isolate 2), C. glabrata (isolate 2), C. parapsilosis, C. dubliniensis, C. guilliermondii, C. albicans (isolate 2), C. albicans (isolate 3), C. albicans (isolate 4), C. tropicalis (isolate 1), C. tropicalis (isolate 2) and C. tropicalis (isolate 3), respectively. [file peerj-09-11082-s003.png]

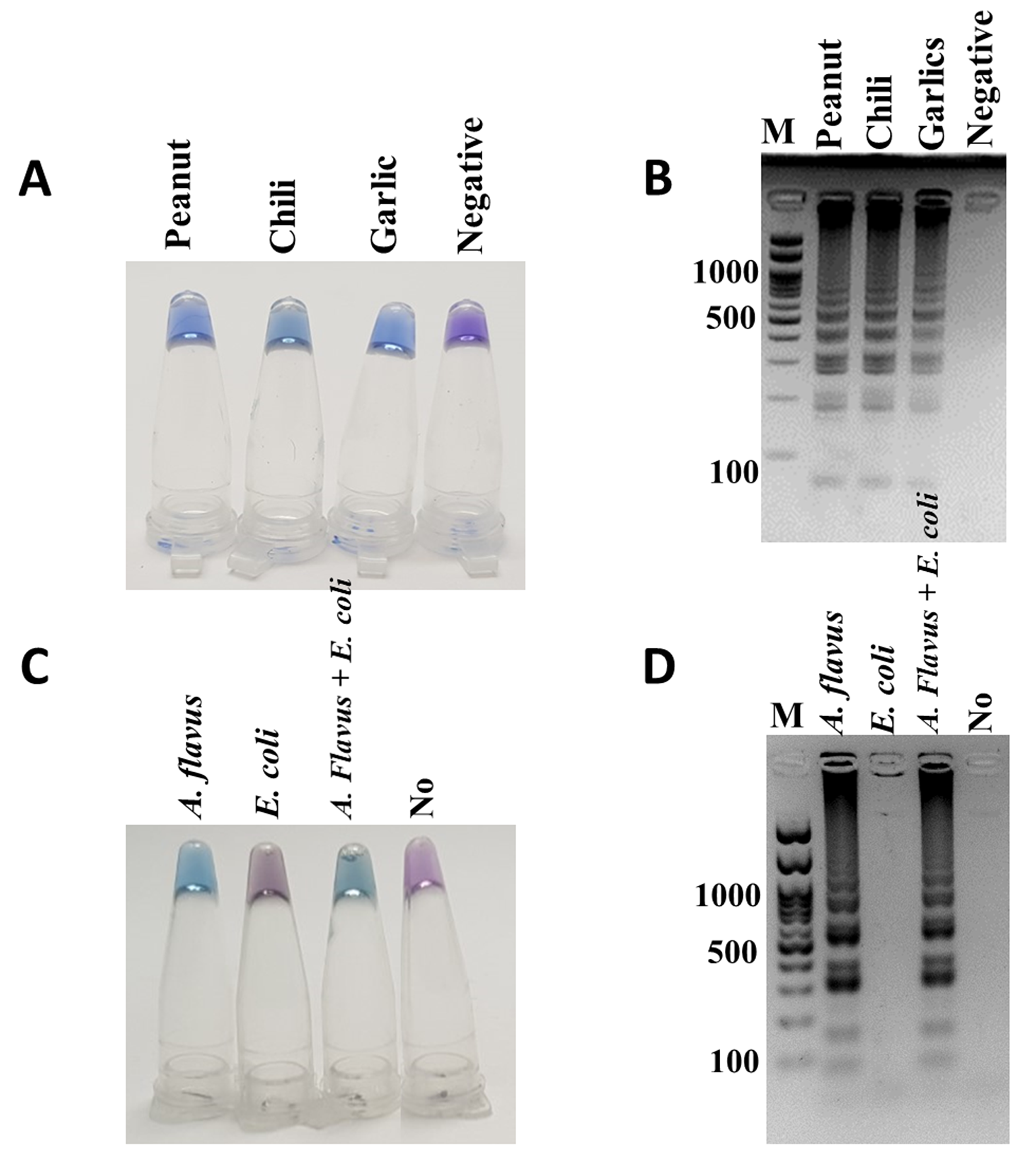

Supplement: Supplemental Information 4 — (A and B) artificially contaminated dry food samples that were tested negative in Fig. 4 by adding 1 pg of A. flavus MSCU0850 culture. (C and D) blood from a healthy volunteer by adding 1 pg each of A. flavus MSCU0850, E. coli, or A. flavus and E. coli cultures, and sterile water (as negative), respectively. [file peerj-09-11082-s004.png]

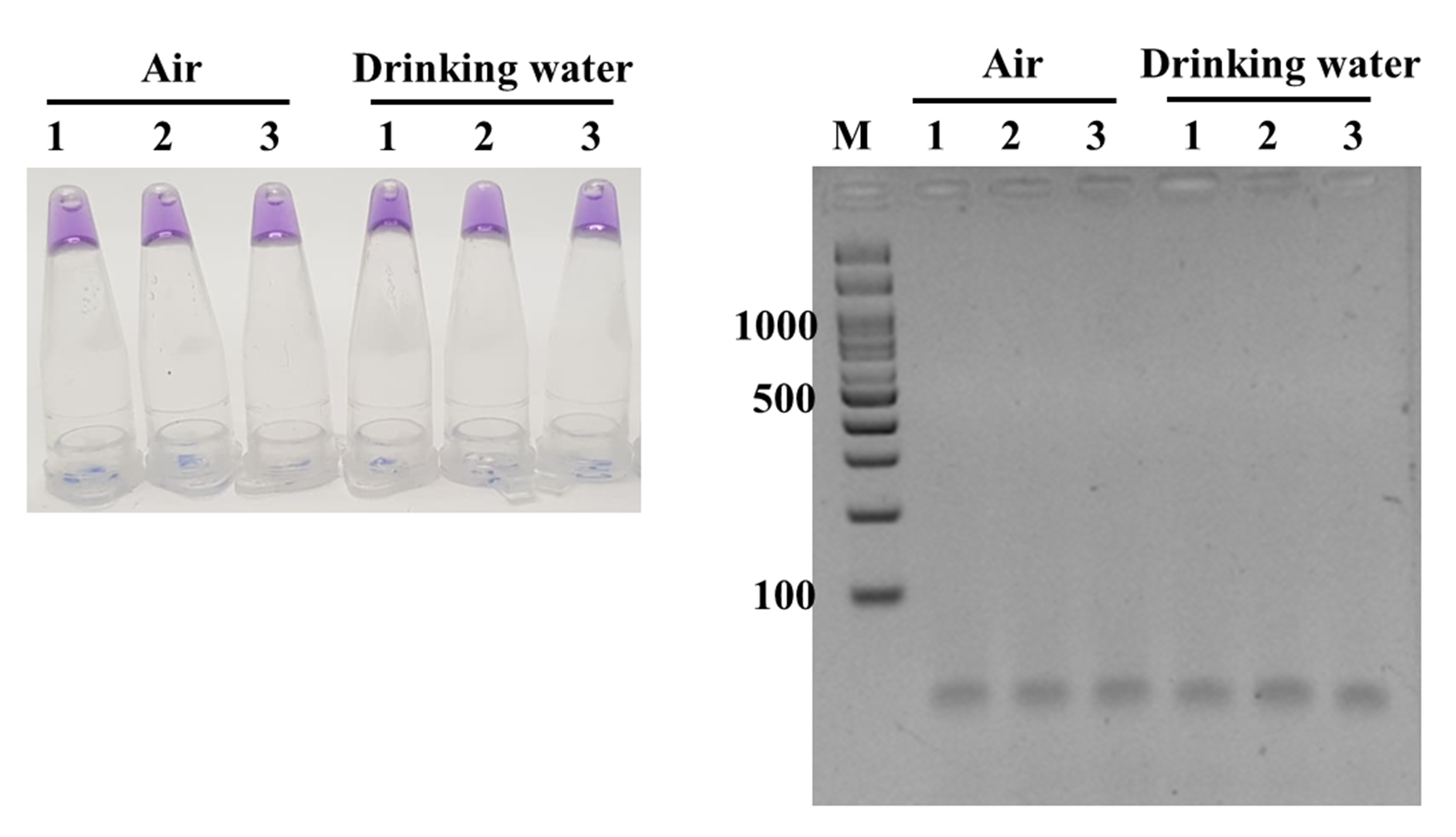

Supplement: Supplemental Information 5 — For air sampling, cotton swab was hold in air in laboratory for 1 min before used in crude DNA extraction. [file peerj-09-11082-s005.png]
