# Supplementary material for: Specific and sensitive, ready-to-use universal fungi detection by visual color using ITS1 loop-mediated isothermal amplification combined hydroxynaphthol blue
Source: PeerJ. 2021 Mar 18;9:e11082. doi: 10.7717/peerj.11082 (PMC7982077; doi:10.7717/peerj.11082)
Supplement: Supplemental Information 7 [file peerj-09-11082-s007.docx]

**Table S1.** List of microorganisms used in this study.

| **Kingdoms** | **Species** | **Strain numbers** | **Sources** |
| --- | --- | --- | --- |
| Fungi | *Rhodotorula mucilaginosa Rhizopus oligosporus Ustilago esculenta* | BCC63208 BCC17810 TBRC1037 | Thailand Bioresource Research Center, BIOTEC, NSTDA |
|  | *Candida albicans Aspergillus carbonarius Aspergillus flavus Curvularia lunata* | MSCU0907 MACU0581 MSCU0850 MSCU0403 | Department of Microbiology, Faculty of Science, Chulalongkorn University |
|  | *Epidermophyton floccosum*  *Trichophyton rubrum*  *Cryptococcus neoformans*  *Microsporum gypseum*  *Flusarium* sp.  *Phialophora verrucose*  *Penicillium marneffei*  *Candida krusei*  *Candida krusei*  *Candida glabrata*  *Candida glabrata*  *Histoplasma* sp.  *Curvularia* sp.  *Candida parapsilosis*  *Candida dubliniensis*  *Candida guilliermondii*  *Candida tropicalis*  *Candida tropicalis*  *Candida tropicalis*  *Candida albicans*  *Candida albicans*  *Candida albicans*  *Candida albicans* | Clinical isolate  Clinical isolate  Clinical isolate  Clinical isolate  Clinical isolate  Clinical isolate  Clinical isolate  Clinical isolate 1  Clinical isolate 2  Clinical isolate 1  Clinical isolate 2  Clinical isolate  Clinical isolate  Clinical isolate  Clinical isolate  Clinical isolate  Clinical isolate 1  Clinical isolate 2  Clinical isolate 3  Clinical isolate 1  Clinical isolate 2  Clinical isolate 3  Clinical isolate 4 | Faculty of Medical Technology, Nakhonratchasima College |
| Bacteria | *Staphylococcus epidermidis Staphylococcus saprophyticus Salmonella typhimurium*  *Escherichia coli*  *Streptococcus pneumonia*  *Proteus mirabilis Staphylococcus aureus Shigella* sp. *Salmonella* sp. *Corynebacterium* sp. *Burkholderia cepacian Acinetobacter baumannii Klebsiella pneumoniae Pseudomonas aeruginosa Enterococcus* sp.  *Neisseria gonorrhoeae Chlamydia trachomatis* | ATCC12228  ATCC15305  ATCC14026  ATCC25922  Clinical isolate  Clinical isolate  ATCC25923 Clinical isolate  Clinical isolate  Clinical isolate  Clinical isolate  Clinical isolate  Clinical isolate  Clinical isolate  Clinical isolate  Clinical isolate  Clinical isolate | Department of Microbiology, Faculty of Science, Chulalongkorn University |
