# Supplementary material for: Specific and sensitive, ready-to-use universal fungi detection by visual color using ITS1 loop-mediated isothermal amplification combined hydroxynaphthol blue
Source: PeerJ. 2021 Mar 18;9:e11082. doi: 10.7717/peerj.11082 (PMC7982077; doi:10.7717/peerj.11082)
Supplement: Supplemental Information 8 [file peerj-09-11082-s008.docx]

**Table S2.** Resolved Fig. 5 equations for days at 50, 75 and 80% ITS1 LAMP activity at 30°C storage, using different preserving additives.

| Preserving additives | Remaining percent of  ITS1 LAMP activity | Days |
| --- | --- | --- |
| 3% glycerol | 80% | 20.08 |
|  | 75% | 23.58 |
|  | 50% | 41.07 |
| 5% glycerol | 80% | 20.03 |
|  | 75% | 23.49 |
|  | 50% | 40.80 |
| 3% PVA | 80% | 22.43 |
|  | 75% | 26.72 |
|  | 50% | 48.16 |
| 5% PVA | 80% | 22.76 |
|  | 75% | 27.13 |
|  | 50% | 48.94 |
